# The Commemoration of Independence Day: Recalling Indonesian Traditional Games

**DOI:** 10.3389/fpsyg.2020.587196

**Published:** 2020-12-10

**Authors:** Mustika Fitri, Hana Astria Nur, Wulandari Putri

**Affiliations:** ^1^Sport Science Study Program, Faculty of Sport and Health Education, Universitas Pendidikan Indonesia, Bandung, Indonesia; ^2^Sport Education School of Postgraduate Studies, Universitas Pendidikan Indonesia, Bandung, Indonesia; ^3^PETE for Elementary School Program, Faculty of Sport and Health Education, Universitas Pendidikan Indonesia, Bandung, Indonesia

**Keywords:** traditional games, traditional sport, cooperation, solidarity, togetherness

## Abstract

Traditional games in Indonesia are one of the cultural heritages the existence of which should be protected and preserved. The purpose of this study was to preserve the cultural diversity by recalling the traditional games through the commemoration of Indonesia Independence Day that is conducted annually. The Online Ethnography method was used in this research by administering text analysis and interview. The result of the study showed that traditional games held during the commemoration of Indonesia Independence Day could help recall the form and the rules of traditional games that are annually conducted during the commemoration in different regions. The result also showed that the traditional games held in the commemoration of Indonesia Independence Day indirectly built a bonding of the people through the value of togetherness, cooperation, and solidarity among the member of the society. Furthermore, the games reflected the cultural diversity of Indonesia, where every region has different names and rules of the traditional games.

## Introduction

Indonesia is a country popular for its diversity and large number of cultures, where every region owns different cultures. In a book published by The Ministry of Education and Culture, the diversity in each region is a social potential that could build the characters and the image of the culture itself, while cultural diversity is an important component in building the image and identity of a culture of a region. Furthermore, diversity is part of intellectual and cultural property of the cultural heritage that should be preserved (Dokhi et al., 2016). It is a responsibility for the society to preserve the existed cultural diversity, while the role of the youth in inheriting the local culture that would be a power for the existence of the local culture itself in the globalization era is highly expected (Nahak, 2019).

Traditional games have developed from a certain habit of a society into a form of game and sport. In the next development, a traditional game becomes a game that owns an original characteristic of a region that is adjusted with the culture of the region. Traditional games are crucial to be protected and preserved for their advantages and cultural values (Anggita et al., 2018). The traditional game in Indonesia is a culture in which the existence should be preserved and retained, as it comes from certain region that has cultural values of society life (Aneka and Rahmatika, 2019). Every region also has traditional games with different names and procedures, although, sometimes, there are games that have similar rules with different names in different regions. As an example, in West Java, there is a game named *galah asin*, while outside West Java, the game is known as *gobak sodor*. Although the name of both of games is different, they have similar rules and procedures. In traditional games, there are games that involve physical activity, known as traditional sports. In relation to the statement, the reconstruction of the traditional game is required so that the game can be implemented and played easily, the participants could understand the value of each game, and, because the traditional games is frequently introduced and played, more children will know and understand the traditional game and its values; thus, the traditional game is automatically preserved (Lintangkawuryan and Adiati, 2017).

According to previous research, traditional games have positive values, including prompting honesty, responsibility for self and others, healthy lifestyle, discipline, hard work, enjoyment, the ability of thinking logically and critically, creativity and innovativeness, rule obedience, respect of others’ duties and achievement, democracy, empathy, awareness on social surrounding, nationalism, social aspect development, and respect of differences. On the other hand, traditional games could also develop friendship aspect, self-management, and academic behavior and decrease antisocial behaviors (Fitria, 2018; Nur et al., 2020).

Among the values of the games, the most distinguished values in the traditional games from Indonesia include the feeling of joy, creativity, sensitivity, social awareness, friendship, and repressing the antisocial behavior. The values in the traditional games could build the togetherness cooperation and familiarity among the people.

In Indonesia, there are traditional games and sports that are regularly conducted during the independence day of Indonesia. There are games for children and adults that are frequently held in different regions, such as *panjat pinang* (slippery pole climbing), *balap karung* (sack race), *gebuk bantal* (pillow fight), *bakiak* (long wooden sandal race), *tarik tambang* (tug of war), and *sepakbola sarung* (playing football with sarong). People are involved in the game enthusiastically. The game is not only fun, but it also reminds and recalls the memory of simple outdoor games that are rarely played by the urban society. When the game is over, the winners usually receive prizes provided by the organizing committee. As argued in an article reported in the Pikiran Rakyat newspaper, the game in Indonesia Independence Day commemoration usually comprises collaboration and togetherness values (Adji, 2018).

In relation to the previous statement, Jabar News states that the competition in the Indonesia Independence Day commemoration, known as *tujuh belas agustusan*, is an annual tradition of the Indonesian people to commemorate the Independence Day; although the games and competition tend to be the same, the people are involved in the game enthusiastically (Rizka, 2019). Therefore, in the written statement of The Ministry of Law and Human Right, cited in tirto.id, the Indonesian people are encouraged to commemorate the independence day by getting involved in activities promoting the value of togetherness, since the activities are conducted to build the spirit of nationalisms and the feeling of love and pride for the country, to strengthen the bonding as Indonesian people, and to show the spirit of cooperation (Dayana, 2019). Unfortunately, there are obstacles in conducting the activities. As reported in merahputih.com, the competitions for commemorating Indonesia Independence Day in the urban area are getting left out, as the place to conduct the activities is limited. Therefore, many regions left the games and activities, although the annual competition has become a tradition in Indonesia and showed the identity of the country (Habib, 2018). It is unfortunate that the games and activities are rarely conducted nowadays, as the commemoration of Indonesia Independence Day has a crucial role. As reported in Jabar News, the presence of social media caused a person to become more individual, while competitions during independence day can be a medium to enable persons to meet in an affordable way, in which a lot of laughing and fun are created and a lot of positive values can be taken from the activities (Rizka, 2019).

A research states that, in Indonesia, especially in Monggak society, the *Engrang* game is still preserved. Although they are also exposed to modernization in reality, but the existence of the competition in commemorating the Independence Day could show the presence of the game. In addition, organizing the competitions could give advantages for the society, including providing a medium to gather up and to interact so that the society could decrease the individualism, embrace the diversity, and maintain the harmony of the Monggak society (Okwita and Sari, 2019).

## Review of Literatures

### Play and Games

Basically, play activity requires willingness, where the players involved in the game could freely choose the game and who they would play the game with. Considering the nature of the game, it will create a comfort and enjoyment for the players. The play activities have a lot of advantages, including improving cognitive aspects, affective aspects, movement skills, social skills, and emotional skills (Rombot, 2017). Regarding the social development aspect, a lot of games involve many people; thus, the game affects the social adaptation and self-adaptation of the players, especially the game involving a social nuance since, in the process, the interaction will grow with both the teammates and the opponents (Gelisli and Yazici, 2015).

A game is a tool to express emotion, interaction, experience, one’s expectation, and the fulfillment of self needs. It is believed to be way to learn. It is also a tool to maintain the body, mind, and character development. The character development is achieved through the evolution in the nature of their game and the change in their social environment (Varzani, 2013).

Games are classified into active and passive games. In active games, the players are required to do a lot of physical activities. Meanwhile, in passive games, the game is for entertainment purpose that does not require a lot of energy. Games consist of two classifications, including individual game and team game. In a team game, the players are divided into groups, small or large. They discuss for their turn to play. In an individual game, the player mostly considers the interest and the winning of themselves; thus, although the game involves many people, individual principles become the first consideration (Hurlock, 2002; Purwaningsih, 2006; Husna, 2009).

### Traditional Sports and Games

Traditional sport and games (TSG) is a specific part of the global sport system that could create the ideas of manifestation of the endangered and exotic culture. It can be analyzed from different perspectives related to sociological science, anthropology, and culture. A game is not only a game, but it is also part of culture. It has a history, an objective, a reason, a structure, a philosophy, and a strategy. It has characteristics and rules, ritual, rhythm, and moral. It connects to a certain environment and has educational dimension that can be learned both from scientific and artistic perspectives. Those perspectives, in short, explain a wider area of research as a cultural study or sport anthropology. Approaches in traditional sports and games can be easily connected to the process of identity that seems to be the main point of TSG role in individual, local, regional, or even national level (Jaouen and Guibert, 2005; Groll et al., 2015).

Defining and classifying TSG is as hard as defining sport in general. It begins with a simple game for children to (semi) professional team sport in a well-organized league or event (Groll et al., 2015). The researchers have been spending efforts in promoting a culture-rich TSG. As argued by a researcher from Japan, through the article in TAFISA magazine, the TSG retention in society is degrading recently. Therefore, Unesco is encouraged to protect and promote this sport to improve the spirit of the community, to keep the society in a togetherness, and to grow the feeling of being proud of the root of the culture of the society (Budde, 2008). All TSGs must have a historical aspect, where they have existed for a long time. Some of the TSGs might have been forgotten, shown by only some persons who play the game. However, in reality, some TSGs still have important roles. The importance of the traditional sport and game is laid on their general characteristics; for instance, TSG is a component of culture and tradition that is important to be preserved and respected for its minimum requirement of tools, simple rules, and also for the type of game that is easy to adapt. TSG recalls us into the cultural diversity and creates the bridge among cultures to acquire a better understanding. Therefore, it is important to preserve and to introduce the traditional sport and games as the heritage of humanity culture and the reminiscence of civilization to be explored in TSG. The concept and the perspective presented originated from sociology and the science of culture that offer a lot of ways to have a further analysis of TSG and their roles in building the bonding among society and creating its identity (Budde, 2008; Groll et al., 2015). It is relevant with the research stating that this sport heritage could help create a harmonious living environment with different cultural expressions and build the bridge among cultures to improve understanding because bonding, value, and practice of togetherness are getting necessary to unite the society. The appreciation of sport and physical activity is a real example. TSG, especially, has been long abandoned in modern life. Therefore, it should be remembered that neglecting the heritage of the traditional sport is the same as eliminating individual culture in a region, a country, or even a continent (Bronikowska et al., 2015).

Furthermore, TSG is also expected to have an active role in the education environment, starting with conducting a teacher training. Nowadays, teachers in various academic levels have not received sufficient training related to traditional games, both in formal and recreational education. Teachers in elementary school and middle school, young bachelor teacher, supervisors in game centers and recreation facilities, and, especially, physical education teachers need a special training to know TSG and to understand their educational values. In some physical education faculties in Europe, traditional sports and games are even taught as part of optional subjects; there is also another initiative such as summer courses offering introduction for 30–40 h of education through traditional games (Lavega, 2006). Moreover, it also has been discussed in the European Traditional Sport and Games Association (ETSGA) forum that, as has been explained by the research of experts, traditional games often offer more possibilities in motor education than the modern sport. Traditional games offer a variety of possibilities, especially with the social–motor collaborative games, but it violates the “image” of inferiority that is usually given to TSG. However, traditional games have another asset to offer; it is the culture diversity in the level of school teaching, where the age group detachment among the generation is increasing. Traditional games offer better socialization, as they do not refer to sports stars. In traditional games, winning is less important; thus, losing the games is also becoming less important (Budde, 2008).

A research on TSG was conducted in Canada. It is especially about an Inuit game that lives and develops in the Nunavut area. Every community promotes the game through schools and recreation centers in their community. The games are integrated into school curricula along with other modern games. There are macro‐ and micro-organizations in the region watching over the development and the organization of the Inuit game. Documents and videos describing the games are present. The Nunanut government financially supports the game. The game is played internationally. The Inuit game begins to appear in the media, but it is still in amateur level where the game is played for fun, fitness, and health. In addition, there is Cree game, but this game does not have the visibility and the success as that of the Inuit game. It might be caused by a lot of variables. The Euro-Canada school system has not been fully aware of the need of the Cree game. The partial Cree Culture curriculum does exist, but it only has a moderate emphasis on the Cree game itself. The school system does not facilitate the integration of the Cree game in school. Cree does not have national nor international competition; thus, the research on the Cree game is still being conducted (Jaouen and Guibert, 2005).

### The Organization and Activities of Traditional Sports and Games

Some traditional sports and games are popular in their area, while others are waiting to be recalled. However, despite the huge differences in popularity, the structure of the organization, and motor complexity, TSG builds an idea where something old, which is geographically specific, is worth to be remembered (Groll et al., 2015). TSGs also relate to the agenda of Sports for All, which is often voiced by The Association for International Sport for All (TAFISA). TSG itself is a source for sports for all; thus, many efforts are administered to realize sports for all through TSG. In Indonesia, there is an organization that accommodates sports for all, namely, the Federasi Olahraga Rekreasi Masyarakat Indonesia (The Federation of Recreation Sports of Indonesia Society), known as FORMI. The organization has joined TAFISA; thus, the existence of FORMI has been recognized internationally. Besides FORMI, Indonesia also has a community named Hong community, founded by Zaini Alif. The community contributes to the preservation of the existence of the traditional games from different regions.

Moreover, the Mindanao people have conducted the sport for all concepts as the inclusive program that do not treat people differently according to their participation advantages in sport and physical activity. Therefore, it should be able to provide opportunities and wider access for all to participate and to respond to the need and interest of the people they serve, including the local people. Sports for All should include a general physical activity that has been long conducted among the local people, including the games and sports, dance, and ritual that come from their neighborhood. In developing culture-based Sports for All for the Mindanao people, they have some stages in the process, including (1) understanding and knowing the people and their culture, (2) having partnership with the community and the leaders, (3) identifying and giving training for the leader of the program, (4) planning the program and the organization, (5) conducting promotion programs and awareness of Sports for All, (6) implementing and supervising the program, and (7) documenting and evaluating the program. The result shows that Sports for All contributes significantly in building healthy cultured people and a strong country (Budde, 2008).

## Research Methods

This present study used an online ethnography method where the research process employed the qualitative approach to collect the data in virtual communities. The value of conducting online ethnography research through internet is not only as a tool but also as conceptual and methodological bridges for other research. The online ethnography research involves activities related to online methods as the practical media in obtaining and collecting data through document collection, online observation, and online interview (Androutsopoulos, 2008; Skågeby, 2011). The data collection technique in this research used the text analysis from different sources, including the media and social media platform such as Instagram and Twitter.

## Discussion

### Recalling Traditional Sports and Games Through the Commemoration of the 74th Independence Day of the Republic of Indonesia

The independence day of the Republic of Indonesia, on 17 August, is often celebrated by conducting various activities followed by the society enthusiastically. One of the activities conducted in celebrating the Independence Day is competition in different places in Indonesia, whether it is in a district level, urban area, national institution, school, or even in a workplace. As cited from an article in iNews.id, “*agustusan* (the celebration of Indonesia Independence Day) is incomplete without competitions, for example *panjat pinang* (slippery pole-climbing), *makan kerupuk* (prawn cracker eating contest), nail in a bottle contest, *gebuk bantal* (pillow fight), or *balap karung* (sack race). The festivity is created when the competitions are conducted. These competitions always invite excitement and laugh” (Teguh, 2019). It is also relevant with a report in tirto.id, “through the competitions in 17th of August, the children are introduced to the struggle and sacrifice of the heroes in struggling for independence. It is drawn in the struggle of the participants to win the competition that is usually called as *tujuhbelasan* competition. Not only for remembering the effort of the heroes, *tujuhbelasan* competition also has a positive advantage for physical and spiritual health” (Dania, 2019). Various typical traditional games, in the Independence Day commemoration, indirectly become the medium to recall the form and the rules of the games. The players indirectly recognize the spirit of struggle and the willingness to struggle in the activities that build the nationalism and independence.

Relevant to the nature of play, which is based on the participant willingness, Respondent 1 says that “the traditional games in *Agustusan* is an entertainment with the purpose of building the spirit and the struggle and creating familiarity.” Thus, the *Agustusan* event gives a social experience containing cooperation, never giving up, and even interaction to raise awareness on the independence that is rarely found in the daily life. The first main point of the statement is that the spirit of struggle in doing any activity with a good mental condition is required. The second point is that the commemoration of the Independence Day becomes an event to recall the traditional games, their rules, and how they are played. It also becomes an event to preserve the games. As cited from an article in Pikiran Rakyat, according to Zaini dewAlif, as the activist of the Hong Bandung community, “August, through the commemoration of the Independence Day on 17 August, becomes a momentum to raise and popularize the traditional sports and games. It is because in August, specifically in the commemoration event, a lot of people, from children to adult, are involved in the traditional games” (Adji, 2018). That is the reason why this annual event is important to be continuously conducted, as through this activity, the society will know and preserve the traditional games and sports of the country.

### The Most Popular Traditional Sports and Games in the Independence Day of the Republic of Indonesia in 2019

On the 74th Indonesia Independence Day, known as *Agustusan*, in 2019, there were various traditional games that the researchers found and analyzed from Indonesian uploads in social media platforms. Respondent 2, related to the commemoration on Indonesia Independence Day, said, “In my opinion, Indonesia Independence Day is one of the moments that we are waiting the most for its fun, familiarity, and togetherness in every series of game conducted.” According to the statement, the respondent rarely feel the moment in daily life. Therefore, the Indonesia Independence Day commemoration becomes important to build positive essentials such as joy, interest, even a strong socialization in various games. Moreover, in the same occasion, Respondent 3 said, “From what I see, the festivity and excitement of the participants are reflected on the games they involved. Hopefully, the joy and togetherness could nourish the warmth among the people in our country, Merdeka!” The statement of Respondent 3 shows that there is an expectation to be noted, especially by an Indonesian citizen; it is preserving the togetherness in maintaining the good name of the country, starting from building a warm atmosphere among individuals. According to Respondent 3, the important point is that the Indonesia Independence Day commemoration becomes the reference in maintaining the warmth among the society in every game conducted. Various games for competitions included *bakiak* race, *balap karung* (sack race), futsal *sarung* (playing futsal with sarong), futsal *daster* (playing futsal with female home dress), *gebuk bantal* (pillow fight), *balap kelereng* (marble race), *makan kerupuk* (prawn cracker eating contest), *panjat pinang* (slippery pole climbing), *tarik tambang* (tug of war), *voli daster* (playing volleyball with female home dress), *voli sarung* (playing volleyball with sarong), crossing the water game, moving the flag game, decorated bicycle parade, nail in a bottle, *egrang* (stilts), *nyunggi tampah*, water collecting game, eel collecting game, coin collecting game, water hitting game, *gobak sodor*, flour pouring game, sandals matching game, leading balloons with *tampah* game, and water pouring game.

*Bakiak* is a kind of sandals made from wood. The *Bakiak* game requires togetherness and cooperation in the team to win. The game prioritizes togetherness and cooperation from the participant. In this game, the participants should walk together to keep the balance and avoid falling. The *bakiak* itself is a long wooden sandal that can be used by four to six participants (Aneka and Rahmatika, 2019). *Balap karung* (sack race) game is one of popular traditional games in Indonesia Independence Day commemoration. To play the game, the participant should put their lower body into a sack; then, they race to the finish line (Munir, 2019). *Balap Karung* games began when Netherlands colonialized Indonesia; this game was often conducted in the institutions and schools built by Netherlands in Indonesia. When Indonesia gained independence, the *Balap Karung* game is preserved by Indonesians, until today, especially in the *17 Agustusan* moment (Widyawati, 2019). Futsal *sarung* game has a similar rule to that of the general futsal game, but in this game, the player should wear a sarong to add more fun to the game. Futsal *daster* game is also similar to futsal *sarung*; the difference is that the player should wear a *daster*, a home dress that is usually used by women at home. *Gebuk bantal* (pillow fight) game is usually conducted above a pond/a river with a long bamboo made like a bridge above the pond/the river. The game includes two participants where the participants hit each other by using a pillow until one of them falls into the pond. The participant who does not fall is the winner. *Balap kelereng* (marble race) game is a game where the participants stand on the start line carrying a spoon filled with a marble on their mouth. When the command is given, they walk as fast as possible to the finish line while keeping the marble on the spoon from falling to the ground without touching the marble nor the spoon.

*Makan kerupuk* (prawn cracker eating contest) game is an individual game, followed by some participants as competitors. The participants should eat a prawn cracker that hangs on a string. The fastest participant who eats up the cracker without using their hands becomes the winner (Husna, 2009; Dewi et al., 2015). In *panjat pinang* (slippery pole climbing) game, an oiled long straight betel palm tree trunk is plugged in on the ground with prizes on the top of the trunk; the participants should climb the trunk to get the prizes; the climber might fall on the climber below them (Damanik, 2019). In this game, the players should help each other. Therefore, one of the members of the team could successfully reach the top of the pole (Yulita, 2017; Damanik, 2019). *Tarik tambang* (tug of war) game is a traditional game that uses a rope with a certain size as a tool to test the strength by pulling the rope, one group at each side of the rope. As with other traditional games, this game is popular in Indonesia (Syukur and Suprayogi, 2016). Similar with futsal *sarung* and futsal *daster*, *voli sarung*, and *voli daster* have similar rules to a general volleyball game. The difference is that the participants wear a sarong or a *daster*.

Crossing the water game generally needs a good balance because, to play this game, every participant should walk on a bamboo above a pond as a bridge. The participant who can pass the bamboo bridge without falling becomes the winner. In moving the flag game, a participant should have at least one opponent, then decides a start line and puts a bottle in a finish line. The participants get ready in the start line while carrying flags; when the game is started, the participants should run as fast as they could to put the flag on the bottle; the first participant who puts the flag on the bottle is the winner (Rasmi, 2017). In the decorated bicycle parade contest, the participant should decorate their bicycle as attractive as possible. When the bicycle is fully decorated, the contestants gather up to join a parade/carnival that has been planned by the committee. The participant with the best/most unique bicycle decoration, according to the jury, becomes the winner. In the nail in the bottle game, the participants will wear a string on their waist with a nail at the tip of the string. The participants get ready at the start line. When the command is given, the participants should reach the bottle at the finish line as fast as possible; then, they focus on putting the nail into the bottle without touching the string nor the nail. *Egrang* (stilts) game is usually played individually or by some participants in groups. The players put their feet on footholds with 50 cm of height from the ground; both of their feet are put on both footholds, and they begin to walk on the *egrang*; the *egrang* itself is usually made from bamboo (Munir, 2019). The *Egrang* game first became popular in West Java. It is the reason why this traditional game becomes the West Java typical traditional game (Sundanese area). The *Egrang* game gains a high interest in other regions in Java because it is fun and interesting (Supriyono, 2018).

In the *nyunggi tampah* game, the participants should make a line at the start line while carrying a *tampah*, a rounded woven bamboo container, on their head. When the command is given, the participants should race to the finish line while keeping the *tampah* from falling without touching the *tampah* itself. The water collecting game is conducted by taking the provided water at the start line; then, they fill the empty bottle at the finish line. The water is brought by using a small glass. The first participant who fills the bottle fully becomes the winner. The Eel collecting game has a similar rule with the water pouring game, but in this game, it is the eels, which have a slippery skin, that should be collected. The participants should take the eel with their bare hands and put the eels into a bottle. The participants who have the highest number of eels will become the winner. In the coin collecting game, some coins will be plugged in an oiled papaya. Half of the coin will appear on the surface of the papaya. As in the prawn cracker eating game, the papaya hangs on a string. The participants should take as many coins as possible. In the water hitting game, some plastic bags are filled with water, tied, and hung on a string. The participants get ready in the start line with their eyes closed. When they hear the command, the participants should walk straightly as fast as possible to hit the water bags hanging on a banana trunk. The participant who successfully smashes the water bag will be the winner.

Gobak sodor game includes two teams. A team consists of three persons or more. The player of the game should stop the opponent from getting into the last line and from going back into the first line, when they first come. To decide the winner, all of the team members should do a back-and-forth process in the field area. The members of the keeper team will keep their area; every person guards the decided line in a zig-zag way. The keeper team should try to get the member of the opponent’s team so that they can have their turn to play the game. Every player who successfully reaches the finish line and gets back into the start line will get one score; the team with the highest score will be the winner (Laelah et al., 2010). There is a unique fact of this game. The name of this game is adapted from the English language “Go Back through the Door.” As the words are hard to be pronounced by the Indonesian people, the name becomes “Gobak Sodor.” Besides, this game came from Yogyakarta, where *gobak* means move freely and *sodor* means spear. This game trains the collaboration among the players and their brain ability in making strategy (Shinta, 2016; Supriyono, 2018). The flour pouring game usually involves two to three teams consisting of four to five persons in a team. Every participant sits in a line with their faces facing forward. The participants hold a plastic plate. The first person in the line holds the plastic plate containing flour. The participants then transfer the flour as fast as possible to the player behind them until the last participant in the line. The group who collects the most flour will be the winner. In the sandals matching game, some sandals are provided randomly. The participant should match a sandal with their pair. The participant who collects most matched sandals will be the winner. The leading balloon with *tampah* game is usually an individual game with some competitors. Every participant holds a *tampah* and a balloon at the start line. When the command is given, the participant leads the balloon to the finish line by using a *tampah*. The participant who reaches the finish line first will be the winner. The water pouring game has a similar rule to the flour pouring game. The difference is on the material. In this game, the material is water, while the media used is a glass.

The researchers found at least 127 posts uploaded by social media users with an open account. From 127 posts, the three most popular games uploaded into social media include *balap karung* (sack race; 27 posts), *bakiak* race (23 posts), and *tarik tambang* (tug of war; 12 posts). From the three most popular games, *balap karung* becomes the most conducted and posted games by social media users. It might be because the rules of the *balap karung* game are often modified; thus, the game often invites laugh for its uniqueness. The modification is usually conducted by adding additional tools. Besides a sack, the participants sometimes use a helmet and blindfold. As cited in Antaranews, “balap karung game with helmet is exciting and challenging. It is also safer than the general *balap karung* game as the participants’ head are protected by the helmet.” “The objective of modifying the *balap karung* game by the committee in commemorating the 74th independence day of Indonesia is to educate the people and participants about wearing a helmet for safety” (Kanafi and Masnun, 2019). Respondent 4 says, “The game seems so exciting. One of the participants was so afraid, that he crawled like a baby rather than jumped. I think the modified *balap karung* game makes the game and the atmosphere lively.” The important point of Respondent 4’s statement is that the games in the Indonesia Independence Day commemoration had essential meanings, such as bravery in facing challenges to achieve the success. The games also created a lively atmosphere for all people, including for men, women, children, youth, and older people. Besides that, the *balap karung* game is interesting and was enthusiastically participated in by the Indonesian Navy and American Navy in the Cooperation Afloat Readiness and Training (Carat) event. As reported in detiknews, “there was much merriment when the *balap karung* game was held. The American navy who had a big body were involved in Indonesia traditional game, that is usually conducted on the Independence Day, without feeling awkward. Even, as they are so powerful, the sack they used was getting broken. Meanwhile, the representative of the US Navy, LT Thomas Cumming, said that he was happy with this sport” (Utomo, 2019). Aside from the merriment, the *balap karung* game could improve self-confidence, especially for children in early childhood (Munir, 2019). The modification in *balap karung* gained attentions from Indonesian people in different regions. This game created a meaningful experience for the players and spectators. The modifications of the game, including using helmet, playing the game in a squat position, playing the game in pair, and even using the blindfold, were accepted enthusiastically by the players. The modifications brought laugh and joy during the game. However, the modification should also consider the safety aspect. Generally, the modification did not change the main rule and important points of the Indonesian traditional game. The existence of the originality of the game enables the society to preserve the local culture principles that would eventually create awareness on the importance of preserving cultures as the identity of the country (Hidayat, 2013).

## Conclusion

One of the promotion efforts to preserve the existence of the games, which slowly disappear and forgotten, is by integrating the games in the activities conducted by organizations related to traditional game and big events regularly conducted in Indonesia, including *Agustusan* held by schools, offices, and institutions in different regions. Some competitions of typical traditional games in the Independence Day commemoration are conducted enthusiastically and full of merriment, although the games are administered annually. In the Agustusan 2019, the most popular traditional game in Indonesia was *balap karung*. It is shown by the number of posts uploaded by Indonesian citizens in social media platforms. Besides, the *balap karung* game became the game that was often modified by the committee of the competitions in different regions.

According to the obtained data, the Indonesia Independence Day commemoration prompted the society to repeat and to recall various traditional sports and games in Indonesia, especially the rules of the game. The moment became an opportunity for Indonesian citizens to preserve the culture and the values of the games. The competition of the traditional games indirectly built the togetherness, cooperation, and familiarity among the people during the activities.

## Author Contributions

MF conducted literature review, analyzed the data, arranged an outline of the draft, supervised the manuscript development, reviewed the manuscript, and gave approval. HN collected and analyzed the data, and wrote the manuscript. WP translated and revised the manuscript. All authors contributed to the article and approved the submitted version.

### Conflict of Interest

The authors declare that the research was conducted in the absence of any commercial or financial relationships that could be construed as a potential conflict of interest.

## References

[ref1] Adji Y. (2018). Mari Hidupkan Permainan Tradisional Saat Perayaan HUT RI 17 Agustus. Pikiran Rakyat. Available at: https://www.pikiran-rakyat.com/jawa-barat/pr-01300290/mari-hidupkan-permainan-tradisional-saat-perayaan-hut-ri-17-agustus-428839?page=2 (Accessed June 25, 2018).

[ref2] Androutsopoulos J. (2008). Potentials and limitations of discourse-centered online ethnography. 5, 1–20.

[ref3] Aneka A. Rahmatika A. (2019). The benefits of traditional game “Clogs” to develop early childhood â€TM s rough motoric. Elementary 5, 107–115. 10.32332/elementary.v5i1.1498

[ref4] Anggita G. M. Mukarromah S. B. Ali M. A. (2018). Eksistensi Permainan Tradisional Sebagai Warisan Budaya Bangsa. J. Sport Sci. Educ. 3, 55–59. 10.26740/jossae.v3n2.p55-59

[ref5] Bronikowska M. Petrovic L. Horvath R. Hazelton L. Ojaniemi A. Alexandre J. (2015). History and cultural context of traditional sports and games in selected European Countries, 1–19.

[ref6] Budde M. (2008). Traditional sport and games: new perspectives on cultural heritage. TAFISA Magazine, 1–68.

[ref7] Damanik M. J. (2019). Sejarah Permainan Panjat Pinang, Penuh Filosofi dan Kontroversi. Available at: https://www.idntimes.com/news/indonesia/margith-juita-damanik/sejarah-permainan-panjat-pinang-penuh-filosofi-dan-kontroversi/4 (Accessed June 27, 2019).

[ref8] Dania R. (2019). HUT ke-74 RI Manfaat Mengikuti Perlombaan 17an. Tirto.id. Available at: https://tirto.id/hut-ke-74-ri-manfaat-mengikuti-perlombaan-17-an-eghP (Accessed June 25, 2019).

[ref9] Dayana A. S. (2019). Tradisi Unik Memperingati HUT RI 17 Agustus di Sejumlah Daerah. Tirto.id. Available at: https://tirto.id/tradisi-unik-memperingati-hut-ri-17-agustus-di-sejumlah-daerah-egef (Accessed July 8, 2019).

[ref10] Dewi S. K. Aziz T. D. U. Susanti A. E. Assagaf L. Muhibba I. dan Nurhasanah (2015). Permainan Tradisional. Jakarta: Kementerian Pendidikan dan Kebudayaan.

[ref11] Dokhi M. Siagian T. H. Sukim Wulansari I. Y. Hadi D. W. Sambodo N. (2016). Analisis Kearifan Lokal ditinjau dari Keragaman Budaya. Jakarta: Pusat Data dan Statistik Pendidikan dan Kebudayaan Kemendikbud.

[ref12] Fitria F. (2018). Development of motorized stimulation models based on traditional Gobak sodor game. 249 (Secret), 87–90.

[ref13] Gelisli Y. Yazici E. (2015). A study into traditional child games played in Konya region in terms of development fields of children. Procedia Soc. Behav. Sci. 197, 1859–1865. 10.1016/j.sbspro.2015.07.247

[ref14] Groll M. Bronikowska M. Savola J. (2015). Cultural aspects of traditional sports and games (September).

[ref15] Habib Z. H. E. (2018). Perayaan 17 Agustus dan Makna di Dalamnya. MerahPutih.com. Available at: https://merahputih.com/post/read/perayaan-17-agustus-makna (Accessed July 8, 2018).

[ref16] Hidayat D. (2013). Permainan Tradisional dan Kearifan Lokal Kampung Dukuh Garut Selatan Jawa Barat. Jurnal Academia Fisip Untad 5, 1057–1070.

[ref17] Hurlock (2002). Psikologi Perkembangan. 5th Edn. Jakarta: Erlangga.

[ref18] Husna (2009). 100 Permainan Tradisional. Yogyakarta: CV. Andi Offset.

[ref19] Jaouen G. Guibert J. (2005). Traditional games: Inheritance, transmission and diffusion. France: Breton Cultural Institute.

[ref20] Kanafi R. I. S. Masnun (2019). Lomba balap karung helm ramaikan HUT RI Ke-74 di Bandarlampung. Antara News. Available at: https://www.antaranews.com/berita/1017130/lomba-balap-karung-helm-ramaikan-hut-ri-ke-74-di-bandarlampung (Accessed June 25, 2019).

[ref21] Laelah F. Nugroho W. Emiliashah B. Karyati E. Maimunah (2010). The character values in traditional game Gobak Sodor for elementary school children, 1–8.

[ref22] Lavega P. (2006). Traditional sports and games in 21st challenges.

[ref23] Lintangkawuryan Y. Adiati M. P. (2017). Recognition of traditional games in Indonesia as cultural preservation efforts through special event. Adv. Econ. Bus. Manag. Res. 28, 216–221. 10.2991/ictgtd-16.2017.42

[ref24] Munir A. (2019). Pengaruh Permainan Balap Karung dan Egrang terhadap Peningkatan Kepercayaan Diri Anak Usia Dini di PAUD Cahaya Kecamatan Rambutan Kota Tebing Tinggi. Jurnal Diversita 5, 161–172. 10.31289/diversita.v5i2.3056

[ref25] Nahak H. M. I. (2019). Upaya Melestarikan Budaya Indonesia di Era Globalisasi. Jurnal Sosiologi Nusantara 5, 65–76. 10.33369/jsn.5.1.65-76

[ref26] Nur H. A. Ma’mun A. Fitri M. (2020). “The Influence of Traditional Games on Social Behavior of Young Millennials.” in *4th International Conference on Sport Science, Health, and Physical Education*; october 8-9, 2019; 251–255.

[ref27] Okwita A. Sari S. P. (2019). Eksistensi Permainan Tradisional Egrang pada Masyarakat Monggak Kecamatan Galang Kota Batam. Historia: Jurnal Program Studi Pendidikan Sejarah 4, 19–33. 10.33373/j-his.v4i1.1720

[ref28] Purwaningsih E. (2006). Permainan Tradisional Anak: Salah Satu Khasanah Budaya yang Perlu Dilestarikan. Jantra: Jurnal Sejarah Dan Budaya I, 40–46.

[ref29] Rasmi L. C. (2017). Lomba Memasukkan Bendera, Perlombaan Simpel yang Penuh Arti. Available at: https://akurat.co/id-58785-read-lomba-memasukkan-bendera-perlombaan-simpel-yang-penuh-arti (Accessed June 27, 2017).

[ref30] Rizka M. (2019). Di Hari Kemerdekaan, Kenali Makna Dibalik Lomba 17 Agustus. Jabar News. Available at: https://jabarnews.com/read/74434/di-hari-kemerdekaan-kenali-makna-dibalik-lomba-17-agustus/1 (Accessed July 8, 2019).

[ref31] Rombot O. (2017). “The application of traditional games to develop social and gross motor skills in 6-7 year-old children” in Proceedings - 2017 International Symposium on Educational Technology, ISET; 27-29 June 2017; Hong Kong, China (IEEE), 116–120.

[ref32] Shinta N. (2016). Asal Usul Gobak Sodor. Tribun Travel, 1–3. Available at: https://travel.tribunnews.com/2016/08/16/asal-usul-gobak-sodor (Accessed October 4, 2016).

[ref33] Skågeby J. (2011). “Online ethnographic methods: towards a qualitative understanding of virtual community practices” in Handbook of research on methods and techniques for studying virtual communities: Paradigms and phenomena (Pennsylvania: IGI Global), 410–428.

[ref34] Supriyono A. (2018). Serunya Permainan Tradisional Anak Zaman Dulu. Jakarta: Badan Pengembangan dan Pembinaan Bahasa, Kementerian Pendidikan dan Kebudayaan.

[ref35] Syukur A. Suprayogi D. (2016). Game Tradisional Tarik Tambang Berbasis Web. IT J. Res. Dev. 1, 38–49. 10.25299/itjrd.2016.vol1(1).673

[ref36] Teguh Z. (2019). 7 Hal Identik dengan Agustusan Nomor 3 paling Seru dan Undang Kemeriahan. iNews.id. Available at: https://www.inews.id/news/nasional/7-hal-identik-dengan-agustusan-nomor-3-paling-seru-dan-undang-kemeriahan (Accessed June 25, 2019).

[ref37] Utomo D. P. (2019). Saat Angkatan Laut AS Jajal Balap Karung dan Lomba Bakiak. detiknews. Available at: https://news.detik.com/berita-jawa-timur/d-4649599/saat-angkatan-laut-as-jajal-balap-karung-dan-lomba-bakiak?utm_content=detikcom&utm_term=echobox&utm_medium=oa&utm_campaign=detikcomsocmed&utm_source=Twitter#Echobox=1564732890 (Accessed June 25, 2019).

[ref38] Varzani M. R. (2013). A study of the role of games in the learning improvement of elementary school boys in Karaj, Iran. Procedia Soc. Behav. Sci. 84, 400–404. 10.1016/j.sbspro.2013.06.574

[ref39] Widyawati R. (2019). Asal Usul Lomba Balap Karung, Kompetisi yang Populer Saat HUT RI ke-74. Tribun Travel, 1–3. Available at: https://travel.tribunnews.com/2019/08/14/asal-usul-lomba-balap-karung-kompetisi-yang-populer-saat-hut-ke-74-ri (Accessed October 4, 2019).

[ref40] Yulita R. (2017). Permainan Tradisional Anak Nusantara. Jakarta: Badan Pengembangan dan Pembinaan Bahasa, Kementerian Pendidikan dan Kebudayaan.

